# Social Media Use and Health and Well-being of Lesbian, Gay, Bisexual, Transgender, and Queer Youth: Systematic Review

**DOI:** 10.2196/38449

**Published:** 2022-09-21

**Authors:** Matthew N Berger, Melody Taba, Jennifer L Marino, Megan S C Lim, S Rachel Skinner

**Affiliations:** 1 Specialty of Child and Adolescent Health, The Children’s Hospital at Westmead Clinical School Faculty of Medicine and Health The University of Sydney Westmead Australia; 2 Centre for Population Health Western Sydney Public Health Unit North Parramatta Australia; 3 Department of Obstetrics and Gynaecology Faculty of Medicine, Dentistry and Health Sciences The University of Melbourne Parkville Australia; 4 Royal Women's Hospital Parkville Australia; 5 Centre for Adolescent Health Murdoch Children’s Research Institute Parkville Australia; 6 Department of Paediatrics Faculty of Medicine, Dentistry and Health Sciences The University of Melbourne Parkville Australia; 7 Centre for Epidemiology and Biostatistics Faculty of Medicine, Dentistry and Health Sciences The University of Melbourne Parkville Australia; 8 Burnet Institute Melbourne Australia; 9 School of Public Health and Preventive Medicine Faculty of Medicine, Nursing and Health Sciences Monash University Melbourne Australia; 10 Kids Research, Children’s Hospital Westmead Sydney Children’s Hospitals Network Westmead Australia

**Keywords:** lesbian, gay, bisexual, transgender, and queer, LGBTQ, adolescence, youth, well-being, mental health, social media, identity, support, mobile phone

## Abstract

**Background:**

Lesbian, gay, bisexual, transgender, and queer (LGBTQ) individuals are at higher risk of poor mental health and well-being. Social media platforms can provide LGBTQ youths with a space that counters heteronormative environments and potentially supports mental health and well-being. Mental health includes an individual’s state of psychological and emotional well-being and not merely the absence of mental disorders.

**Objective:**

We sought to identify how LGBTQ youths and adolescents use social media for connection with other LGBTQ peers and groups, identity development, and social support and how these affect mental health and well-being.

**Methods:**

PRISMA (Preferred Reporting Items for Systematic Reviews and Meta-Analyses) procedures were used to guide this review. Searches were conducted in ACM Digital Library, CINAHL, Ovid Embase, Ovid MEDLINE, and Web of Science in March 2021. This review focused on LGBTQ youths aged 10 to 24 years. Included peer-reviewed studies must comprise social media; explore peer connection, identity development, or social support; and be published from 2012 onward. In total, 2 researchers extracted data and performed quality assessments independently using the Newcastle-Ottawa Scale for quantitative articles and the Critical Appraisal Skills Programme for qualitative articles. Qualitative synthesis was performed on articles that satisfied the eligibility criteria.

**Results:**

A total of 26 studies (n=15, 58% qualitative; n=8, 31% quantitative; n=3, 12% mixed methods) met the inclusion criteria. Of the 8 quantitative studies, 6 (75%) were cross-sectional, and 2 (25%) were cohort studies. All studies ranged from moderate to high quality. Social media was a popular tool used by LGBTQ youths to connect with LGBTQ communities. In qualitative data, we found that LGBTQ youths negotiated and explored identity and obtained support from peers on social media. Instagram, Tumblr, and Twitter were commonly used to access LGBTQ content owing to ease of anonymity. Identity management was the most studied social media affordance, important to LGBTQ youths for strategic disclosure. Key strategies for managing identities included being anonymous, censoring locations or content, restricting audiences, and using multiple accounts. Quantitative studies (3/8, 38%) showed that social media was associated with reduced mental health concerns and increased well-being among LGBTQ youths. Mental health concerns arising from social media use were attributed to discrimination, victimization, and policies that did not accommodate changed identities.

**Conclusions:**

We found that social media may support the mental health and well-being of LGBTQ youths through peer connection, identity management, and social support, but findings were limited by weaknesses in the evidence. More robust and longitudinal studies are needed to determine the relationship between social media use and LGBTQ mental health, particularly among adolescents. The findings may inform interventions to promote social media health literacy and the mental health and well-being of this vulnerable group.

**Trial Registration:**

PROSPERO CRD42020222535; https://www.crd.york.ac.uk/prospero/display_record.php?RecordID=222535

## Introduction

### Background

Recent years have seen social media become a part of our daily lives, especially for adolescents and young adults [1]. Facebook, Instagram, Snapchat, TikTok, and YouTube are among the most popular platforms used by adolescents [2,3]. Social media use can be defined as web-based behaviors using platforms to like, comment, message, or monitor other users [4]. Social media can be used to overcome barriers of distance and expand or consolidate web-based communities [5]. Several benefits to well-being have been associated with social media, including strengthened peer relationships, involvement in specific social networks, and facilitation of identity expression [6,7]. Social media platforms are constantly evolving and encourage a plethora of activities ranging from communicating with family and friends to sharing content and knowledge [8]. Motivations for social media use include entertainment, relationships, information, and identity development and management [9]. Lesbian, gay, bisexual, transgender, and queer (or questioning; LGBTQ) people are heavier users of social media and are more likely to have multiple accounts compared with their non-LGBTQ counterparts [10].

LGBTQ people experience higher rates of mental health concerns and behaviors, including suicidal ideation, self-harm, anxiety, depression, and posttraumatic stress disorder [11]. LGBTQ populations are also at a higher risk of experiencing violence, discrimination, and adversity [12-14]. LGBTQ youths in particular have a higher prevalence of victimization than non-LGBTQ youths because of increased exposure to prejudice and violence at school [13]. Unsupportive family and peers contribute significantly to an increased risk of mental health disorders and substance use [15-17]. However, in some situations, disclosure of sexual or gender identity, or “coming out,” is associated with reduced mental health issues [15-17]. To counter the negative consequences of coming out, some LGBTQ individuals use selective disclosure strategies, particularly because of concerns about losing friends or family [16]. Family, friend, and society acceptance are associated with better mental health, well-being, and self-esteem in LGBTQ individuals [18,19]. Other support networks such as involvement in LGBTQ sporting clubs can also improve mental health and well-being among LGBTQ people [20,21]. In addition, LGBTQ people tend to rely on other LGBTQ individuals for support [10,20,21], and not connecting with LGBTQ support networks is associated with poorer mental health outcomes [20].

Many LGBTQ individuals live in environments where sexuality and gender diversity are not accepted [11]. At least 69 countries criminalize same-sex relationships, and 9 countries criminalize gender nonconformity [22]. These environments make LGBTQ identity development difficult and, in public, individuals are forced to conform to heteronormativity to avoid persecution. Even societies that are more accepting of LGBTQ people maintain mainstream heteronormative environments [23]. For example, school sex education focuses on heterosexual people and is rarely inclusive of other sexualities [24,25]. Social media can act as a safe environment to access information about identity, express identity, or provide support among LGBTQ people, thus supporting mental health and well-being [26-34]. Although individual studies have shown benefits, there has not been a review of studies to synthesize the range of benefits. It is important to understand how social media is used by LGBTQ youths to explore their identities and connect with like-minded people and how this affects mental health. Thus, this highlights the important role that social media plays regarding LGBTQ youth for policy makers, educators, and clinicians working in this area. This population is an important focus for research because of the increased risk of compromised mental health and well-being [11].

### Aims

In this systematic review, we sought to examine studies exploring the relationship between social media use and mental health and well-being among LGBTQ youths. Specifically, we aimed to identify how LGBTQ youths and adolescents use social media for (1) connection with other LGBTQ peers and groups, (2) identity development, and (3) social support and how these affect mental health and well-being. We also sought to identify any impact of social media on the mental health of LGBTQ youths. The World Health Organization classifies young people as those aged between 10 and 24 years [35]. For the purpose of this review, “youth” includes adolescent and youth ages.

## Methods

### Registration and Search Strategy

This review was registered with PROSPERO before data synthesis (CRD42020222535; Multimedia Appendix 1). Electronic databases were searched for literature, including CINAHL (1939; March 2021), Ovid Embase (1947; March 2021), Ovid MEDLINE (1946; March 2021), Web of Science (1900; March 2021), and ACM Digital Library (1985; March 2021). Additional studies were found through Google Scholar and PubMed and added to the screening process. A hand search of the reference lists of the included papers was also conducted to identify any studies missed in the search terms. Individual database searches are listed in Multimedia Appendix 2. These searches were conducted using a search strategy with the following keywords: LGB* or GLB* or Sexual and Gender Minorities or gay or lesbian or queer or transgender or sexually and gender diverse or gender and sexually diverse or homosexual* or bisexual* or sexual orientation AND identit* or support* or help* or friend* or relationship* or partner* or mental health or depression or anxiety or mood disorder or posttraumatic stress disorder or PTSD or suicid* or self-harm or wellbeing AND social media* or social networking site* or Facebook or Instagram or Tumblr or Twitter* or YouTube or LinkedIn or WeChat or Snapchat or TikTok AND adolescen* or young adult* or teen* or youth*.

### Inclusion and Exclusion Criteria

To be included, studies needed to (1) have a sample consisting of at least 50% individuals aged 10 to 24 years to ensure that the study focused on youths; (2) be specific to LGBTQ populations or present LGBTQ findings separately from any non-LGBTQ sample; (3) include social media use as a predictor; (4) explore connecting with peers, identity development, or social support; (5) be published from 2012 onward to capture recent forms of social media platforms and use (including smartphone apps); and (6) be available in full text and in English. All study designs were eligible, including quantitative, qualitative, and mixed methods research. Only peer-reviewed articles of original research were eligible; case studies, narratives, conference presentations, and other nonempirical works were not included. Papers were first screened by title and abstract (961/1234, 77.88%) and again by full text (101/961, 10.5%) by MNB and MT. A total of 26 papers were appraised by MNB and MT. Disagreements during title and abstract screening and full-text assessments were discussed between MNB and MT, and any disagreements were resolved through team discussion.

### Quality Assessment

All the included studies were subject to quality appraisal to assess the research design, ethics compliance, and risk of bias. The Newcastle-Ottawa Quality Assessment Scale (NOS) was used to assess the quality of quantitative studies [36], including an adapted version for cross-sectional studies [37]. The NOS assesses studies based on 3 domains: selection, comparability, and outcome [36]. For qualitative studies, the Critical Appraisal Skills Programme (CASP) was used to assess quality [38]. Both the NOS and CASP were applied to mixed methods studies. Mixed methods studies are discussed in the relevant qualitative or quantitative sections.

### Data Synthesis

The PRISMA (Preferred Reporting Items for Systematic Reviews and Meta-Analyses) procedures were used to guide the review (Multimedia Appendix 3) [39]. A quality assessment table displaying CASP or NOS scores and a summary table of the studies, including study characteristics, were produced. As quantitative studies used different measures for outcomes and took different statistical approaches, a meta-analysis was not possible. For qualitative data, a predefined schema was developed to assist with data collection based on the review’s aims before conducting the search according to preliminary literature searches. Included qualitative data were thematically synthesized according to the 3 stages of Thomas and Harden [40], namely, coding, developing and refining themes, and generating analytical themes. The findings were divided into 3 themes and, within each theme, into qualitative and quantitative findings. The three themes as developed by the schema were (1) connecting with other LGBTQ youths on social media, (2) LGBTQ identity development using social media, and (3) social support on social media. For this review, “queer” represents gender or sexualities otherwise not classified within lesbian, gay, bisexual, and transgender. Identity development refers to the exploration of a diverse sexuality or gender, which includes discovery, awareness, appraisal, communication, and how individuals manage their identity [41]. Social support via social media among LGBTQ youths refers to receiving assistance and feeling cared for [26]. These are important factors with the potential to promote mental health and well-being when individuals can explore and connect in safe spaces [42].

## Results

### Overview

**Figure 1:**
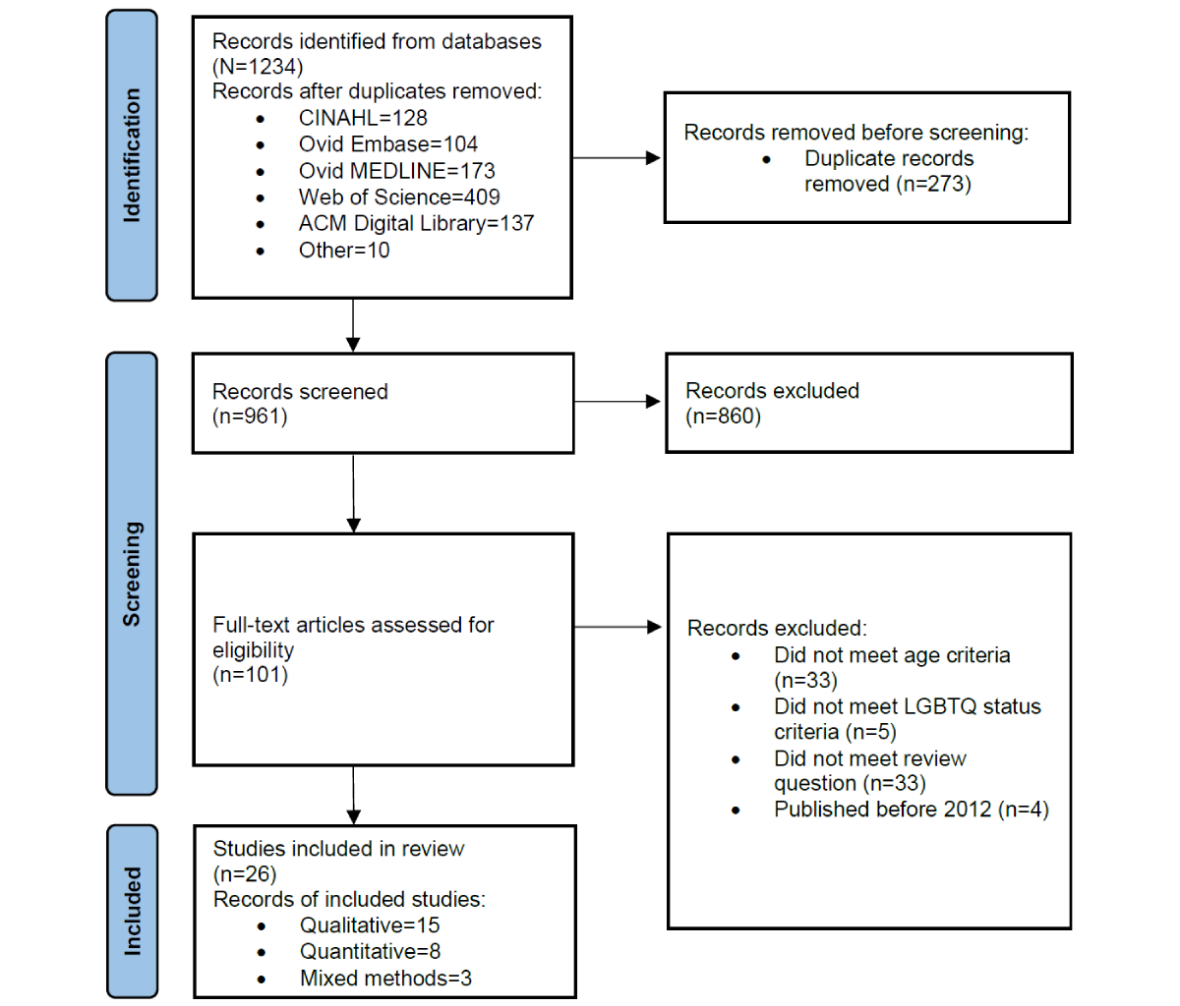
PRISMA (Preferred Reporting Items for Systematic Reviews and Meta-Analyses) flow diagram of the selection process. LGBTQ: lesbian, gay, bisexual, transgender, and queer.

This search resulted in a total of 961 papers retrieved from the specified databases, with 273 (28.4%) duplicates removed (Figure 1). Title and abstract screening excluded 89.5% (860/961) of the papers, leaving 101 papers for full-text screening, of which 26 (25.7%) met the aims and criteria of this review. Of the 26 included papers, 15 (58%) were qualitative studies, 8 (31%) were quantitative studies, and 3 (12%) were mixed methods studies. The included studies were mostly conducted in the United States (17/26, 65%), whereas others were conducted in Australia (2/26, 8%), Canada (4/26, 15%), China (1/26, 4%), Ukraine (1/26, 4%), and the United Kingdom (3/26, 12%). The ages of the study participants ranged from 13 to 34 years, with a total of 14,112 participants across the 26 studies. One study appeared to meet the inclusion criteria, but we were unable to confirm the age descriptions of their sample through the full text or contacting the authors [23]. Summaries of quality assessments are provided in Table 1, and summaries of included qualitative and quantitative studies are provided in Table 2 and Multimedia Appendix 4.

**Table 1:** Quality assessment summaries and limitations of the included studies (N=26).

Study, year	CASP^a^ score	NOS^b^ score	Comments and limitations
Bates et al [27], 2020	8/10 criteria	N/A^c^	Generalizability: Participants predominately White and openly LGBTQ^d^ All recruited from 1 university
Bond and Figueroa-Caballero [43], 2016	N/A	8/10 stars	Cross-sectional study Generalizability: Data collected from gay-straight alliances
Byron et al [31], 2019^e^	8/10 criteria	5/10 stars	Generalizability: Race and ethnicity not well described Internal validity: Possible risk of interviewer bias not described
Ceglarek and Ward [44], 2016	N/A	9/10 stars	Cross-sectional study Internal validity: Data were self-reported, which may be prone to social desirability or recall bias Inadequately validated measures
Chong et al [45], 2015	N/A	7/10 stars	Cross-sectional study Generalizability: Small Hong Kong–based LGB^f^ population
Craig and McInroy [32], 2014	9/10 criteria	N/A	Generalizability: Most participants were from progressive, well-educated, and affluent backgrounds
Craig et al [46], 2021	N/A	6/10 stars	Cross-sectional study Internal validity: Inadequately validated measures Sample characteristics not described (covariates)
Duguay [47], 2016	8/10 criteria	N/A	Generalizability: Small gender-diverse population within the sample All participants were university students
Fox and Ralston [48], 2016	9/10 criteria	N/A	Generalizability: Participants predominately White From 1 city in the United States, with most being college students Internal validity: Possible risk of interviewer bias not described
Hanckel et al [49], 2019	7/10 criteria	N/A	Generalizability: Suboptimal description of race and ethnicity but indicating a lack of diversity Internal validity: Selection and recruitment not described Analytical method unclear
Harper et al [33], 2016	10/10 criteria	N/A	Generalizability: Data were collected from 2004 to 2006 and, thus, may not represent current use and past perceptions of LGBTQ identities Only recruited from 2 metropolitan US cities Limited ethnic backgrounds because of the parent study aims
Herrera [50], 2018	8/10 criteria	N/A	Generalizability: No participant characteristics described Limited to lesbian or queer-identifying women Limited to Instagram use Internal validity: Concern of selection and interviewer bias as the investigators invited participants to interviews by commenting on Instagram posts
Hillier et al [28], 2012	9/10 criteria	N/A	Generalizability: Participants predominately White
Lucero [29], 2017^e^	8/10 criteria	5/10 stars	Generalizability: Small sample of Ukrainian youths that may not be representative of the Ukrainian population Investigators reported their results as LGBTQ although there were no transgender participants Internal validity: Significantly small sample size for quantitative analysis Inadequately validated measures
McConnell et al [51], 2018^e^	10/10 criteria	6/10 stars	Generalizability: From a single metropolitan US city The disclosed LGBTQ identity cohort were likely overrepresented because of the significantly higher sample size compared with nondisclosed cohorts Participants predominately African American
McConnell et al [52], 2017	N/A	6/10 stars	Prospective cohort study Generalizability: Participants predominately African American Internal validity: Loss to follow-up not described
McInroy et al [30], 2019	N/A	7/10 stars	Cross-sectional study Internal validity: Possible selection bias as the study aimed to compare on the web and offline; however, it recruited primarily on the web
McInroy and Craig [53], 2015	8/10 criteria	N/A	Generalizability: From a single metropolitan Canadian city Small transgender subpopulation Internal validity: Most had high motivation or knowledge of media and may be associated with volunteer bias
Paceley et al [54], 2020	9/10 criteria	N/A	Generalizability: From small towns or rural areas in 1 US state Although they purposefully recruited diverse participants, there was limited intersectional analysis Underrepresentative of transgender population
Pellicane et al [55], 2020	N/A	5/10 stars	Prospective cohort study Generalizability: Undergraduate psychology students from 1 university Participants predominately female Internal validity: Strong risk of volunteer bias because of selection Loss to follow-up not described
Rubin and McClelland [34], 2015	9/10 criteria	N/A	Generalizability: Most from a single metropolitan US city Internal validity: Small sample size
Selkie et al [56], 2020	9/10 criteria	N/A	Generalizability: From 1 gender services clinic in the Midwestern United States All had supportive parents because of recruitment from the clinic Internal validity: Participants’ locality not collected (eg, rural or metropolitan)
Singh [57], 2013	9/10 criteria	N/A	Internal validity: Risk of bias from telephone interviews
Taylor et al [58], 2014	10/10 criteria	N/A	Generalizability: Underrepresentative of transgender population
Twist et al [59], 2017	N/A	4/10 stars	Cross-sectional study Generalizability: Undergraduate students minoring in Family Studies from 1 university Internal validity: Strong risk of volunteer bias because of selection Small sample size Data were self-reported, which may be prone to social desirability or recall bias
Varjas et al [60], 2013	9/10 criteria	N/A	Generalizability: Owing to age, parental permission was required, and the study likely included only those whose parents knew and were supportive

^a^CASP: Critical Appraisal Skills Programme.

^b^NOS: Newcastle-Ottawa Quality Assessment Scale.

^c^N/A: not applicable.

^d^LGBTQ: lesbian, gay, bisexual, transgender, and queer.

^e^Mixed methods studies.

^f^LGB: lesbian, gay, and bisexual.

**Table 2:** Summary of the included quantitative studies (N=11).

Study, year, and country	Purpose	Age (years)	Sample size, N	LGBTQ^a^ sample	Method	Findings	Summary
Bond and Figueroa-Caballero [43], 2016, United States	Understand the relationships among technology, sexual identity, and well-being based on age, gender, geographic location, race, and religion	13 to 19 (mean 16.5, SD 1.3)	570	Gay (45%), bisexual (27%), and lesbian (24%)	Recruitment: from gay-straight alliances and web-based message boards; data collection: questionnaires and surveys; measures: Rosenberg Self-Esteem Scale, Multiple Affect Adjective Check List, Multidimensional Scale of Perceived Social Support, and Measure of Sexual Identity Exploration and Commitment	Using regression analyses, the study found that LGB^b^ youths spend more time on social media compared with non-LGB youths, with time spent significantly on sexual identity (*β*=.14; *P*<.05) and well-being (*β*=.11; *P*<.05). Time spent on social media was associated with sexual identity (*β*=.08; SE 0.02; *P*<.001) but not directly with well-being (*β*=.04; SE 0.03; *P*=.21). Well-being was significantly associated with sexual identity commitment (*β*=.47; SE 0.08; *P*<.001).	Social media demonstrated a connection with sexual identity development associated with well-being. LGBTQ youths used social media to understand sexuality and give social support, which may not be as significant offline.
Byron et al [31], 2019, Australia	How Tumblr is used among LGBTQ youths to connect with peers and develop identity and well-being	16 to 34 (mean 24.6)	1304	Homosexual (33.9%); bisexual (24.7%); queer (18%); and pansexual, agender, panromantic, and demisexual (19.8%)	Recruitment: via social media advertisements and flyers to LGBTQ organizations; data collection: semistructured interviews and questionnaires and surveys; measures: 2 nominal questions	Tumblr was the platform that participants most often left (11.7%; excluding Myspace and Tinder). Tumblr was abandoned for several reasons: 34% found it too time-consuming, 30% felt it became a negative space, and 15% found it to have negative health impacts.	Negative experiences were common, with participants describing Tumblr as becoming toxic, although it was useful.
Ceglarek and Ward [44], 2016, United States (Michigan)	Understand LGB use of social media for identity exploration and expression and connecting with LGB communities	18 to 24 (mean 20.23, SD 1.68; LGBTQ participants)	570	Heterosexual (n=446), homosexual (n=68), not sure (n=4), and other (n=21)	Recruitment: from LGBTQ support organizations; data collection: questionnaires and surveys; measures: Lesbian, Gay, and Bisexual Identity Scale as well as the Short Scale for Measuring Loneliness in Large Surveys and the Brief Symptom Inventory	Among LGB youths, higher social support on social media was associated with lower levels of loneliness (*β*=−0.27; *P*≤.01) and paranoia (*β*=−0.21; *P*≤.05) using *β* coefficients. There were no significant differences for anxiety (*β*=−0.03; *P*>.05), depression (*β*=−0.10; *P>*.05), hostility (*β*=−0.04; *P*>.05), and sensitivity (*β*=−0.04; *P*>.05). Learning about sexuality via social media reduced anxiety (*β*=−0.35; *P*>.05), hostility (*β*=−0.32; *P*≤.05), and paranoia (*β*=−0.43; *P*≤.01).	Social media has potential to allow LGBTQ youths to develop identity and, thus, have improved mental health. When seeking identity expressions and social support, the web may provide avenues with reduced stigmatization compared with offline.
Chong et al [45], 2015, China (Hong Kong)	Understand LGB social media use for identity, community monitoring, and support and sense of belonging	Mean 23.3 (SD 6.33)	233	Lesbian (n=86), gay (n=107), and bisexual (n=40)	Recruitment: flyers distributed to LGBTQ organizations and social media; data collection: questionnaires and surveys; measures: Inclusion of Community in Self Scale, Mental Health Inventory, Life Satisfaction Scale, and Satisfaction with Life Scale	Using structural equation modeling, sense of belonging among LGB youths was associated with social media use for LGB group membership (*β*=.22; *P*<.05). LGB group connection via social media was indirectly associated with improved mental well-being through reduced stigma (*β*=.27; *P*<.05). Social media use to enhance LGB connection and reduce stigma affected mental well-being (*β*=.06 and 0.09; *P*<.05).	Social media is a vital resource for LGB youths to express sexual or gender identity and social support. Mental health can be improved with positive social media capital.
Craig et al [46], 2021, Canada and United States	Explore benefits of social media among LGBTQ youths and develop the Social Media Benefits Scale	14 to 29 (mean 18.21, SD 3.6)	6178	Pansexual (n=1782), bisexual (n=1602), queer (n=1305), gay (n=970), lesbian (n=968), asexual (n=691), not sure (n=398), cisgender (n=3950), gender nonconforming (n=2168), and transgender (n=909)	Recruitment: flyers displayed on the web on social media and sent to LGBTQ organizations; data collection: questionnaires and surveys; measures: Social Media Benefits Scale	Of those who chose Facebook as their favorite platform, 11% reported that it helped them feel loved. Adolescents (aged 14-18 years) were the most likely group, and those aged 19 to 24 years were the second most likely group, to use social media for emotional support and development (*F*=75.88; *P*<.001).	Younger youths were more likely to use social media for its benefits, such as social support, connectivity, and information. Youths would commonly connect with LGBTQ individuals or groups and celebrities. Other benefits included improved emotional support and development.
Lucero [29], 2017, Ukraine and United States	Examine whether social media provides LGBTQ youths with a safe space for identity exploration and expression	14 to 17 (mean 16.3)	19	Lesbian (n=3), gay (n=8), bisexual (n=1), queer (n=1), unsure (n=3), and not straight (n=3)	Recruitment: flyers sent to LGBTQ organizations and Facebook; data collection: questionnaires and surveys; measures: Social Media Frequency Survey and Facebook Intensity Scale	Three-quarters of Facebook users never or rarely experienced cyberbullying and considered it a safe space for connecting and communicating with others. Over two-thirds of participants reported social media to be a comfortable environment compared with offline.	LGBTQ social media users felt safe to communicate and explore with peers on platforms such as Facebook.
McConnell et al [51], 2018, United States (Chicago)	Examine the relationship between Facebook and LGBTQ youth identity management	19 to 28 (mean 24.13, SD 1.64)	199	Identifying as male (n=77), identifying as female (n=108), transwomen (n=15), transmen (n=3), gay (n=69), lesbian (n=55), bisexual (n=49), heterosexual (n=10), and unsure (n=8)	Recruitment: LGBTQ youths from a longitudinal study; data collection: questionnaires and surveys; measures: adapted Outness Inventory	Participants were grouped into 4 categories of level of identity disclosure on Facebook: cluster 1 (high overall outness), cluster 2 (low overall outness), cluster 3 (less out to family), and cluster 4 (more out to family). Cluster 1 comprised 64% of the participants with high levels of disclosure among family, classmates or colleagues, and others.	LGBTQ youths felt that free self-expression on social media was complicated because of factors relating to identity disclosure. By investigating Facebook accounts, youths were mostly categorized as being of either low or high outness. Some would purposely censor their identity expression to avoid unintentional identity disclosures.
McConnell et al [52], 2017, United States (Chicago)	Examine Facebook use among LGBTQ youths, identity management methods, and effects of outness	Mean 24.02 (SD 1.65)	204	Transgender (n=24), gay (n=59), lesbian (n=49), bisexual (n=42), heterosexual (n=9), and unsure (n=5)	Recruitment: LGBTQ youths from a longitudinal study via email and flyers sent to LGBTQ organizations; data collection: questionnaires and surveys; measures: adapted Outness Inventory, Multidimensional Scale of Perceived Social Support, and Brief Symptom Inventory	Over 13% had multiple Facebook accounts, and >42% used privacy settings to limit viewable content for selected friends. Participants reported high outness offline and on Facebook, both positively correlated (*r*=0.72; *P*<.001). Facebook outness showed a high positive correlation (*r*=0.73) and the lowest correlation among friends (*r*=0.53).	Social media can act as a strategy for identity management, which some users find important. Some LGBTQ youths possessed multiple accounts or platforms where they could differ identity expression according to audience.
McInroy et al [30], 2019, Canada and United States	Explore LGBTQ engagement with web-based and offline communities, activities, and resources	14 to 29 (mean 18.35, SD 3.64)	4009	LGBTQ+ (n=7986), heterosexual (n=58), and cisgender (n=2211)	Recruitment: from LGBTQ organizations and school groups; data collection: questionnaires and surveys; measures: 6-scale questionnaire on activeness, support, and safety in web-based and offline LGBTQ communities	LGBTQ participants would connect more with the LGBTQ community on the web (88%) compared with offline (69%). LGBTQ participants were more engaged (2-tailed t_4008_=10.12; *P*<.001) and supported (t_4008_=26.28; *P*<.001) and safer (t_4008_=35.78; *P*<.001) on the web compared with offline. LGBTQ social media or blogs were used by 87% of the participants, and identity-specific web or YouTube series were used by 79% of the participants.	LGBTQ youths were likely to participate on the web with other LGBTQ people, including social media. Social media was reported to be a safer, more supportive, and more active option compared with offline.
Pellicane et al [55], 2020, United States (Midwest)	Examine relationships between social media acceptance and hostility and their effects on mental health	Mean 19.87	387	Heterosexual (n=326), bisexual (n=40), homosexual (n=7), and other (n=5)	Recruitment: undergraduate psychology students from an electronic database; data collection: questionnaires and surveys; measures: Center for Epidemiological Studies-Depression Scale, State-Trait Anxiety Inventory, and Social Media Experiences Questionnaire	There were significant associations between acceptance via social media and reduced symptoms of depression (*β*=−0.453; *P*<.001). Higher social media acceptance was also significantly associated with reduced anxiety symptoms (*β*=−0.343; *P*<.001). Conversely, hostility on social media was associated with increased symptoms of depression (*β*=.120; *P*=.19).	Social media has the benefit of acceptance and support for LGBTQ individuals and can help prevent or reduce anxiety and depression. This pattern was not reflected among the non-LGBTQ population in this study.
Twist et al [59], 2017, United States (Southwest)	Explore LGB experiences of monitoring web-based visibility and relationships	18 to 41 (mean 24.67)	61	Bisexual (n=33) and same-sex orientated (n=28)	Recruitment: undergraduate students; data collection: questionnaires and surveys; measures: Lesbian, Gay, and Bisexual Identity Scale as well as the Ecological Elements Questionnaire, Family Adaptability and Cohesion Scale-IV, and Same-Sexting Practices and Questionnaire	Facebook had high levels of visibility regarding LGB identity, relationship disclosure (32%), gender identity (30%), and sexuality (31%). Almost half (49%) of the participants felt that partner outness on the web was immaterial. Most (70%) reported infrequent negative responses to web-based identity disclosure.	Most participants reported their sexual identity via social media primarily on Facebook. Most participants did not report negative interactions because of their identity disclosure on social media.

^a^LGBTQ: lesbian, gay, bisexual, transgender, and queer.

^b^LGB: lesbian, gay, and bisexual.

### Quality Assessment

Overall, the included articles were moderate to high in quality and limited to descriptive study designs. Both CASP and NOS scales ranged from 0 to 10. Qualitative data were of high standard, with a mean of 8.7 (SD 0.8, range 7-10), whereas quantitative data were of moderate standard, with a mean of 6.2 (SD 1.5, range 4-9). Half of the qualitative studies (9/18, 50%) were limited by a lack of sample description [31,33,49,50,56], having predominately White samples [27,28,32] with higher socioeconomic backgrounds [32], or having a small sample size [34]. A total of 6% (1/18) of the qualitative studies had only 8 participants [34], but the sample size was not included in the CASP. The importance of sample sizes in qualitative research to data adequacy is an open question [61]. A total of 6% (1/18) of the studies collected data from 2004 to 2006 [33]. In total, 11% (2/18) of the studies had small transgender subpopulations (n≤4) [53,54]. A total of 11% (2/18) of the studies were limited because of their restrictive recruitment (ie, primarily from 1 source) [27,48]. A total of 6% (1/18) of the studies recruited participants from a gender diversity clinic requiring parental permission, thus introducing bias by selecting youths with more supportive parents [56]. Of the 11 quantitative studies, 7 (64%) used cross-sectional designs [29-31,45,47] with a low [43,44] to medium risk of bias [29,59]. A total of 9% (1/11) of the studies used a longitudinal design but provided no information on loss to follow-up [55]. Only 18% (2/11) of the studies described the assessment and management of confounding [43,44]. Of the 11 studies, 4 (36%) had generalizability concerns [29-31,48], 2 (18%) had insufficient descriptions of the sample [31,46], 2 (18%) used inadequately validated measures [29,46], and 1 (9%) had significant volunteer bias [30]. A total of 9% (1/11) of the studies were limited because of the small sample size (n=19) [29].

### Connecting With Other LGBTQ Youths on Social Media

#### Overview

Qualitative (13/18, 72%), quantitative (3/11, 27%), and mixed methods (2/3, 67%) studies found that web-based environments were safe spaces for LGBTQ peer connection [27-31,33,34]. Qualitative data on LGBTQ youth social media use consisted of subthemes, including that anonymity is used to connect with peers, youths connect differently depending on the social media platform, and social media reduces feelings of isolation. LGBTQ youths commonly connected with peers via social media platforms [27,31,32,54,60]. All the included studies (26/26, 100%) were at risk of volunteer bias because of the nature of the target population and recruitment methods.

#### Qualitative Studies

A total of 72% (13/18) of the qualitative studies explored narratives about LGBTQ youths’ connection with peers via social media [27,28,32-34,49-51,53,54,56,57,60]. Instagram, Tumblr, Twitter, and YouTube were commonly used to connect, at times anonymously [31,49,51,54,56]. A total of 8% (1/13) of the studies, which found that Facebook policies limited anonymity, were weakened by the fact that they did not specify an analysis method or describe recruitment [49]. Tumblr was popular among LGBTQ youths, providing community connection, information, and support [31,49,54]. Participants reported that they ceased using Tumblr once it became “toxic” and negatively affected their mental health [31]. Instagram users were able to find and connect with others via hashtags (eg, #lesbian), although it should be noted that this study was limited by selection biases as recruitment occurred by inviting participants through Instagram comments [50]. LGBTQ youths could cease negative interactions (eg, block profiles) easily via social media if they felt uncomfortable talking to others [54].

LGBTQ youths also resorted to social media to connect with the LGBTQ community when there was a lack of offline opportunities [34,54]. Social media was a vital tool for those in rural and remote settings to connect with LGBTQ peers [33]. Youths reported reduced feelings of isolation and increased well-being when connecting with other LGBTQ youths [33,54,56,60]. LGBTQ youths could converse with LGBTQ peers anonymously and, as comfort increased, meet offline [33,49,54]. The study by Varjas et al [60] required parental permission, which limited their sample to those with generally supportive parents. Developing a web-based and offline connection with those who shared the same identities helped form emotional connections within the community and between individuals (eg, romantic relationships) [28,33,34,54]. These platforms also acted as a mechanism for LGBTQ youths to engage in sexual encounters on the web or offline [54]. LGBTQ youths were more likely to meet their web-based connections in person compared with non-LGBTQ peers [28]. Many LGBTQ youths turned to web-based spaces such as social media as their offline environment was unaccepting [28,54].

#### Quantitative Studies

A total of 45% (5/11) of the studies investigated peer and group LGBTQ connections among youths [29-31,45,46]. A total of 20% (1/5) of these studies reported that 65% of 1304 LGBTQ Tumblr users in Australia used the platform to connect with other LGBTQ youths [31]. Only 3% of the participants used Tumblr to connect with friends; rather, it was specifically used to interact with strangers who shared their identities [31]. Social media was used to connect with others, including LGBTQ celebrities or groups that improved their sense of belonging and provided gratification [30,45,46]. A total of 20% (1/5) of the studies noted that approximately 80% of 6178 LGBTQ youths followed LGBTQ celebrities and communities [46]. In total, 20% (1/5) of the studies identified that mental health and well-being were positively affected by social media connection, but this study was limited because of its small sample size of 19 adolescents, mainly gay men (42%) [29].

### LGBTQ Identity Development Using Social Media

#### Overview

All study designs explored this theme (qualitative: 16/18, 89%; quantitative: 4/11, 36%; mixed methods: 1/3, 33%). This theme explored LGBTQ youth identity development and management through the use of social media strategies for identity expression, accessing information, and censorship. Subthemes included the use of anonymity and privacy settings, sharing and validating identity development experiences, and the disclosure of identity. These strategies focused on methods to avoid conflict and protect well-being [27,34,51,57]. Healthy identity development can improve mental health and well-being among LGBTQ youths [43,44,49].

#### Qualitative Studies

Nearly all qualitative studies (16/18, 89%) explored concepts of LGBTQ identity development via social media [27,28,32-34,47-51,53,54,56-58,60]. Studies noted from participant narratives that Facebook, Tumblr, and Twitter tended to be used more than other platforms for facilitating identity development [27,28,31,49,54,58]. LGBTQ youths found social media vital for identity development as it reduced the danger and stigma of meeting in person [32,58]. LGBTQ youths developed understanding and acceptance of and comfort with their identity through exposure to experiences of peers via forums, videos, and written blogs [32,33,53,54,56]. Social media allowed these individuals to explore their identities safely and access gender identity transition information [28,32,48,54,56,57]. A total of 6% (1/16) of these studies had a risk of bias because of the use of telephone interviews only and a small sample size (n=13) [57].

Facebook, Tumblr, and Twitter were commonly mentioned platforms that facilitated identity expression and exploration [27,31,49,58]. Many turned to Tumblr and Twitter to specifically express their LGBTQ identity rather than Facebook because of its restrictive policies and audiences (ie, changing the name in the profile’s URL and limited identity options) [31,49,58]. Young people found that connecting with LGBTQ communities allowed them to share experiences, for example, medical information and surgery experiences for transgender youths [33,48,53,56,60]. Many appreciated sharing feelings and lived experiences, reporting that other LGBTQ individuals understood them better compared with non-LGBTQ people [33,50]. Narratives from participants included how social media can be a safe environment that facilitates healthy identity development because of privacy setting features imperative to LGBTQ youths’ web-based engagement [27,32,47,58]. Privacy settings and “friending” practices provided them with the ability to choose their social network audience and, therefore, how they expressed their identity [27,47,50]. This permitted LGBTQ youths to manage disclosure experiences such as gradually disclosing one’s identity or remaining undisclosed if preferred [27,47].

Disclosing identity on the web provided the user with time to consider and articulate how they would communicate their identity to their offline networks [33]. Social media platforms such as Facebook Messenger allow gender-diverse users to change their nicknames to suit their identity, which could aid in gradual identity disclosure [28,49]. For some, it was vital to remain undisclosed to avoid danger, relationship deterioration, and negative interactions [47]. Social media could offer identity disclosure without the expectations, danger, and pressure associated with offline networks [28,32]. LGBTQ youths could express their identity by sharing with their audience using subtle posts (eg, images of same-sex partners, pronouns, names, and relationship statuses) [27,32,47,54,58].

Others reported that, if sexual preferences were left empty on Facebook, the person was considered likely not heterosexual [34,47]. Less subtle displays of “outness” usually occurred by having highly expressive and visible profiles [47]. These actions required considerable contemplation of the potential repercussions and reactions of audiences [32,51]. Social media offered a way for LGBTQ youths to disclose their identity without reprisal from friends or family [32]. Social media distanced LGBTQ youths from heteronormative environments, homophobia, and transphobia that they may have experienced offline [32,49,51,53,56,57]. There were mixed views of the platforms’ (ie, Facebook’s) use of LGBTQ-specific categories, with some praising the understanding of their identity and others finding it restrictive (eg, interested in men or women and other pre-existing terminology) [27,48]. A study focusing on Instagram users found that using identity hashtags was a better way to connect with peers [50].

Qualitative studies noted that having multiple social media accounts permitted LGBTQ youths to express and explore identities with specific audiences with anonymity [27,31-33,47,49-51]. Family, religious groups, and work were commonly named as audiences with whom LGBTQ youths needed multiple accounts and self-censorship to manage [51,58]. Pressure was experienced as friends and family monitored LGBTQ youths’ social media [34,49]. Accidental disclosure of an LGBTQ identity, most commonly by sharing with unintended audiences, was identified as a risk of social media for identity expression [47,49,51]. Preventative strategies, which are often successful, included separating audiences, deidentifying locations and names, and adjusting privacy settings [47,49,51]. These strategies assisted in managing exposure to marginalization and stigma [49]. Even when censoring identity on social media, other indicators such as likes, images, group memberships, and friends’ posts and events could be displayed [34,47]. Constantly monitoring and censoring references to LGBTQ content to avoid negative interactions could be overwhelming and cause youths to conform to heteronormative expectations [51].

Being able to view and interact with others expressing similar LGBTQ identities was validating for youths [33,53]. Seeing other youths, including schoolmates, engaging in LGBTQ-orientated activities on social media allowed for further identity exploration and understanding [33]. This exposure to other LGBTQ youths helped affirm one’s identity and prove that LGBTQ people exist (eg, “liking” posted LGBTQ content) [28,33,50,51,53,56,57]. Shared backgrounds were another important factor for identity affirmation among ethnic minorities and religious groups [33,57,58]. Social media may assist in identity clashes (ie, LGBTQ and Christian identities) that create difficulties in understanding, exploration, and transition [58].

#### Quantitative Studies

A total of 55% (6/11) of the studies examined LGBTQ identity development and management [29,43,44,51,52,59]. Overall, an increased understanding of identity via social media was associated with improved well-being outcomes [43,46]. Social media was reported as a safer and more comfortable approach for identity exploration than offline alternatives [29]. A study among same-sex attracted youths in the United States noted that 63% of 61 participants had their identity deliberately disclosed on social media [59]. This study had significant generalizability issues as recruitment was restricted to undergraduate students of 1 degree at 1 university [59]. Identity exploration and well-being were associated with higher use of social media among lesbian, gay, and bisexual (LGB) youths compared with non-LGB youths attending a straight-gay alliance at a US high school [43]. Identity exploration via social media was associated with lower paranoia scores among American LGB youths [44]. However, the heavy use of social media for identity exploration had negative mental health consequences, increasing loneliness and sensitivity to emotional, physical, or social stimuli [44].

In a study of American LGBTQ youths, 13% of 181 participants had multiple Facebook accounts for identity exploration or expression [52]. Of this sample, 27% had publicly visible profiles, whereas 54% restricted their profiles to friends [52]. In total, 43% restricted what their friends could view on their Facebook profiles [52]. High levels of disclosure on Facebook were common, with 64% of 199 LGBTQ participants freely displaying their identity [51]. Another study found that 30% of LGBTQ youths disclosed on Facebook, significantly higher than on other platforms, including Tumblr (5%-9%) or Twitter (8%-13%) [44]. The level of identity disclosure related to the individual’s willingness to express their identity [51]. LGBTQ youths who were not disclosed to their family were often highly engaged with and disclosed to their LGBTQ networks on the web compared with those who were disclosed to their family [51]. LGBTQ youths did not consider their partners not being disclosed on social media or offline as an issue for their relationship or satisfaction, with 23 of 61 participants reporting that it was “extremely unimportant” [59].

### Social Support on Social Media

#### Overview

This final theme explored the support mechanisms that LGBTQ youths used via social media (qualitative: 5/18, 28%; quantitative: 4/11, 36%; mixed methods: 2/3, 67%). Subthemes in the qualitative data on LGBTQ youths using social media included seeking social support from peers and communicating information and experiences between peers. LGBTQ youths would find support by connecting with other LGBTQ people or groups and obtaining pertinent information [28,33,53,54,56]. Access to social support and information can be beneficial for mental health and well-being [46,55].

#### Qualitative Studies

Almost half (7/18, 39%) of the qualitative studies explored social support among LGBTQ youths through social media [28-31,33,54,56]. Social support among LGBTQ youths was more commonly reported as occurring on the web compared with non-LGBTQ youths, whose offline networks were sufficient [28]. Social media connections were useful for seeking support during difficult times for young LGBTQ individuals [28,54,56]. Facebook was used to participate in LGBTQ groups where individuals could express emotions and seek support [29,54].

Web-based friends could provide support without geographical restrictions as participants communicated with others in different countries [54,56]. Posting on social media about mental or physical health concerns was not always a method to elicit social support but, rather, to simply be heard [31]. LGBTQ youths were able to interact with other or experienced LGBTQ community members for advice on dating, safety, sex, identity disclosure, and sexuality [28,33,54]. Social support via social media was highly convenient and could be obtained whenever required, even at short notice [54]. Transgender youths were able to seek specific support from other transgender individuals and share transition experiences [53,56]. Many transgender youths reported viewing YouTube videos as support for their transition as such information was generally inaccessible offline [53]. Transgender youths were able to access pertinent medical information and resources [53].

#### Quantitative Studies

A total of 45% (5/11) of the studies examined social support through social media among LGBTQ youths [31,44-46,55]. Social media afforded LGBTQ youths support that they might not have achieved offline [46]. Ceglarek and Ward [44] reported that, among their 570 participants, there was strong evidence (*β*=−0.27; *P*≤.01) that the use of social media for support was linked to reduced loneliness and evidence (*β*=−0.21; *P*≤.05) of reduced paranoia among LGB youths from self-reported data. There was weak or no evidence (*P*>.05) that social media use among LGB youths reduced anxiety and depression [44]. Social media acceptance and support were associated with reduced symptoms of depression among 387 LGB youths (*β*=.453; *P*<.001) but had no significant effect on anxiety; however, this study did not describe loss to follow-up [55]. Social media use was associated with feelings of being loved or feeling stronger [45]. Although Tumblr was not a uniformly positive experience, 30% of 1304 surveyed LGBTQ youths reported it as a useful resource [31]. As age increased, the use of social media for social support and information among 6178 participants decreased significantly (*P*<.001) [46].

### Discussion

#### Principal Findings

Similar systematic reviews have been published previously [62,63], but this is the first to explore how LGBTQ youths use social media and its impact on mental health and well-being. This systematic review explored how LGBTQ youths use social media and how it affects their peer connections, identity exploration, and social support. We found 26 studies—overall, the quality of the research was moderate and limited to observational studies. Most studies (16/26, 62%) were limited by a lack of follow-up, limited description of study confounders, restrictive sample eligibility limiting generalizability, and selection biases. Causality cannot be inferred from associations. With these limitations in mind, how LGBTQ youths use social media for connection and identity has been well explored. Both the positive and negative impacts of social media use among LGBTQ youths were identified.

We identified multiple ways in which social media has a positive impact on the mental health and well-being of LGBTQ youths. The studies showed a reduction in mental illness symptoms, including anxiety, depression, and paranoia [34,44,45,55,60]. Participant narratives identified decreased feelings of isolation and increased well-being when engaging in social media [33,54,56,60]. Social media was a significant source of social support for LGBTQ youths [28,33,54,56]. It was a setting where young people could control the expression of their sexual and gender identities to prevent or reduce exposure to stigma and discrimination [27,28,32-34,47-51,53-55]. Alleviating stressors among LGBTQ youths is associated with a reduced risk of poor mental health, including depression and suicidal ideation [43]. Other literature has found that developing networks and expressing LGBTQ identity safely (without stigma or discrimination) leads to reduced mental health problems, including anxiety, depression, addictive behaviors, and suicidal ideation [11,64].

We also identified the negative outcomes of social media use. Heavy social media use among LGBTQ youths was associated with increased feelings of loneliness and sensitivity [44]. Social media dependency was also linked to poorer academic performance, sleep deprivation, and mental health conditions [62,65]. Although social media could limit discrimination and stigma, LGBTQ youths are still at higher risk of web-based victimization [66], and other research has noted that social media can be a source of discrimination, including within web-based LGBTQ networks [26]. Mental health and well-being were negatively affected by social media structures and policies that did not accommodate changed identities [49]. A study found that chosen identity recognition may be associated with reduced mental illness symptoms [67]. Much of the wider literature identifies the effects of social media among LGBTQ youths as generally negative [52]. However, this review identifies both the positive and negative aspects of social media use among LGBTQ youths. Some platforms (eg, Grindr, Tinder, and Twitter) offer users nonbinary options, but displaying this information could also lead to safety issues [47,68]. Being “outed” on social media can target LGBTQ youths for physical harm and discriminatory comments, with the potential to affect their mental health and well-being [55]. Although a study exploring the use of hashtags only found positive and negative outcomes of identity exploration [50], another study found that hashtags can be used for other purposes, such as connecting peers that use drugs (eg, #highlife) [69].

We found that social media allowed LGBTQ youths to actively manage their identity, whereas non-LGBTQ youths did not demonstrate the same use of social media or the need to explicitly express their identity [27,34,50,51,58]. LGBTQ youths would actively manage their audiences by friending those of similar ages, limiting some of them via privacy settings, or removing friends [27,47]. These platforms’ affordances made it possible for LGBTQ youths to connect with numerous other LGBTQ people and disclose their identity regardless of physical location [33,54,70]. In contrast, non-LGBTQ individuals reported sufficient support offline and did not add strangers to their social media [28].

This review noted that Facebook and Twitter had higher identity disclosure than other platforms and sexuality-specific dating apps [59]. Web-based sexual encounters usually occur via geosocial networking apps that allow for location sharing among LGBTQ and non-LGBTQ individuals (eg, Grindr, Tinder, and Bumble) [54,71,72]. Other social media platforms such as Facebook were also used for this purpose but less commonly [73]. Connections among LGBTQ individuals or communities can lead to romantic relationships, overcoming barriers such as fewer potential romantic partners and societal restrictions [11]. Dating same-sex partners was associated with improved mental health and self-esteem and reduced internalized homophobia compared with dating other sex partners [11]. The wider literature found that, for young people, negative aspects of social media included jealousy (eg, images of their partner with other people) or inaccurate social media depictions of relationships [74,75]. LGBTQ relationship portrayals and web-based engagement on social media may also affirm one’s identity [28,33,50,51,53,56,57]. It is important to note that social media landscapes can change rapidly in platform popularity, as seen in recent years with the gradual decrease in Tumblr use and the rise of TikTok [76,77].

#### Limitations

Only published peer-reviewed data (and no gray literature) were included. This systematic review was also limited as the sensitive nature of sexual and mental health meant that individual studies were at risk of reporting bias. There were very few studies (5/26, 19%) that investigated social media use influences on the mental health of LGBTQ youths. In addition, there was no uniform measure assessing mental health outcomes to determine the effect of social media use on mental health. Finally, owing to the ever-changing nature of social media and digital technology, these concepts may not capture current experiences.

#### Conclusions

This review identified LGBTQ youths’ uses of social media to connect with like-minded peers, manage their identity, and seek support. In the few studies that considered mental health outcomes (5/26, 19%), the use of social media appeared to be beneficial to the mental health and well-being of this group [11,34,44,55,60]. In this systematic review, we identified the various important beneficial roles of social media, but the findings were limited by weaknesses in the evidence base. This information may be useful for professionals (eg, educators, clinicians, and policy makers) working with LGBTQ youth to consider the appropriate use of social media in interventions as it provides an evidence base for the role of social media in the lives of LGBTQ youths. These findings help further understand how LGBTQ youths use social media and its positive and negative impacts on their mental health and well-being. Further research is required to provide stronger evidence of how social media is used for connectivity, identity, and support and determine causal links to mental health outcomes. We recommend larger, representative, and prospective research, including intervention evaluation, to better understand the potential of social media to support the health and well-being of marginalized LGBTQ young people. It is imperative that social media is understood and its beneficial use is supported to ensure improved outcomes.

## Appendix

Multimedia Appendix 1 PROSPERO registration.

Multimedia Appendix 2 Search strategy.

Multimedia Appendix 3 PRISMA (Preferred Reporting Items for Systematic Reviews and Meta-Analyses) checklist.

Multimedia Appendix 4 Summary of the included qualitative studies (N=18).

## Abbreviations

CASP: Critical Appraisal Skills Programme
LGB: lesbian, gay, and bisexual
LGBTQ: lesbian, gay, bisexual, transgender, and queer
NOS: Newcastle-Ottawa Quality Assessment Scale
PRISMA: Preferred Reporting Items for Systematic Reviews and Meta-Analyses
