# Supplementary material for: Social Media Use and Health and Well-being of Lesbian, Gay, Bisexual, Transgender, and Queer Youth: Systematic Review
Source: J Med Internet Res. 2022 Sep 21;24(9):e38449. doi: 10.2196/38449 (PMC9536523; doi:10.2196/38449)
Supplement: Multimedia Appendix 1 [file jmir_v24i9e38449_app1.pdf]

Systematic review

*Fields that have an **asterisk (*)** next to them means that they **must be answered. Word limits** are provided for each section. You will be unable to submit the form if the word limits are exceeded for any section. Registrant means the person filling out the form.*

This record cannot be edited because it has been marked as out of scope

2. Original language title.

For reviews in languages other than English, give the title in the original language. This will be displayed with the English language title.

3. * Anticipated or actual start date.

Give the date the systematic review started or is expected to start.

02/12/2020

4. * Anticipated completion date.

Give the date by which the review is expected to be completed.

01/03/2021

5. * Stage of review at time of this submission. [2 changes]

This field uses answers to initial screening questions. It cannot be edited until after registration.

Tick the boxes to show which review tasks have been started and which have been completed.

Update this field each time any amendments are made to a published record.

The review has not yet started: No

Review stage	Started	Completed
Preliminary searches	Yes	Yes
Piloting of the study selection process	Yes	Yes
Formal screening of search results against eligibility criteria	Yes	Yes
Data extraction	Yes	Yes
Risk of bias (quality) assessment	Yes	Yes
Data analysis	Yes	Yes

Provide any other relevant information about the stage of the review here.

7. * Named contact email.

Give the electronic email address of the named contact.

9. Named contact phone number.

Give the telephone number for the named contact, including international dialling code.

12. * Review team members and their organisational affiliations.

[1 change]

Give the personal details and the organisational affiliations of each member of the review team. Affiliation refers to groups or organisations to which review team members belong. **NOTE: email and country now MUST be entered for each person, unless you are amending a published record.**

Mr Matthew Berger. University of Sydney
Ms Melody Taba. University of Sydney
Dr Megan Lim. Burnet Institute
Dr Jennifer Marino. University of Melbourne
Professor Rachel Skinner. University of Sydney

13. * Conflicts of interest.

List actual or perceived conflicts of interest (financial or academic).

None

14. Collaborators.

Give the name and affiliation of any individuals or organisations who are working on the review but who are not listed as review team members. **NOTE: email and country must be completed for each person, unless you are amending a published record.**

16. * Searches.

State the sources that will be searched (e.g. Medline). Give the search dates, and any restrictions (e.g. language or publication date). Do NOT enter the full search strategy (it may be provided as a link or attachment below.)

CINAHL, Embase, MEDLINE, Web of Science and ACM Digital Library.

18. * Condition or domain being studied.

Give a short description of the disease, condition or healthcare domain being studied in your systematic review.

The potential for improved mental health/prevention of mental health issues among the vulnerable group of LGBTQ youth.

20. * Intervention(s), exposure(s).

Give full and clear descriptions or definitions of the interventions or the exposures to be reviewed. The preferred format includes details of both inclusion and exclusion criteria.

Youth are frequent users of social media platforms (e.g. Facebook, Snapchat and Instagram) which has many functions that apply to youth's daily life. For LGBTQ youth this may support wellbeing and belongingness, and without social media these youth may be at risk for social and physical isolation effecting their mental health.

22. * Types of study to be included.

Give details of the study designs (e.g. RCT) that are eligible for inclusion in the review. The preferred format includes both inclusion and exclusion criteria. If there are no restrictions on the types of study, this should be stated.

Only peer-reviewed articles of original research will be deemed eligible; case studies, narratives, conference speeches and other non-empirical works are not included.

24. * Main outcome(s).

Give the pre-specified main (most important) outcomes of the review, including details of how the outcome is defined and measured and when these measurement are made, if these are part of the review inclusion criteria.

Notable differences in wellbeing, belongingness and general mental health of those LGBTQ youth who use/used social media to seek/provide support and make connections via social media.

This can include youth perceptions/experiences that determine increased feeling of wellbeing and belongingness or changes in odds ratios in favour of social media reducing negative mental health outcomes among LGBTQ youth.

26. * Data extraction (selection and coding).

Describe how studies will be selected for inclusion. State what data will be extracted or obtained. State how this will be done and recorded.

• [illegible] least one other co-author

28. * Strategy for data synthesis.

Describe the methods you plan to use to synthesise data. This but should be and describe how the proposed approach will be applied to your data. If meta-analysis is planned, describe the models to be used, methods to explore statistical heterogeneity, and software package to be used.

A qualitative synthesis will be conducted to address the review question using the SPIDER model. (1) See below for reference.

30. * Type and method of review. [1 change]

Select the type of review, review method and health area from the lists below.

Type of review

Cost effectiveness
No

Diagnostic
No

Epidemiologic
No

Individual patient data (IPD) meta-analysis
No

Intervention
No

Living systematic review

No

Meta-analysis
No

Methodology
No

Narrative synthesis
No

Network meta-analysis
No

Pre-clinical
No

Prevention
No

Prognostic
No

Prospective meta-analysis (PMA)
No

Review of reviews
No

Service delivery
No

Synthesis of qualitative studies
No

Systematic review
Yes

Other
No

Health area of the review

Alcohol/substance misuse/abuse
No

Blood and immune system
No

Cancer
No

Cardiovascular
No

Care of the elderly
No

Child health
No

Complementary therapies
No

COVID-19
Yes

For COVID-19 registrations please tick all categories that apply. Doing so will enable your record to appear in area-specific searches

Chinese medicine
Diagnosis
Epidemiological
Genetics
Health impacts
Immunity
Long COVID
Mental health
PPE
Prognosis
Public health intervention
Rehabilitation
Service delivery
Transmission
Treatments
Vaccines
Other

Crime and justice
No

Dental
No

Digestive system
No

Ear, nose and throat
No

Education
No

Endocrine and metabolic disorders
No

Eye disorders
No

General interest
No

Genetics
No

Health inequalities/health equity
No

Infections and infestations
No

International development
No

Mental health and behavioural conditions
Yes

Musculoskeletal
No

Neurological
No

Nursing
No

Obstetrics and gynaecology
No

Oral health
No

Palliative care
No

Perioperative care
No

Physiotherapy
No

Pregnancy and childbirth
No

Public health (including social determinants of health)
No

Rehabilitation
No

Respiratory disorders
No

Service delivery
No

Skin disorders
No

Social care
No

Surgery
No

Tropical Medicine
No

Urological
No

Wounds, injuries and accidents
No

Violence and abuse
No

32. * Country.

Select the country in which the review is being carried out. For multi-national collaborations select all the

countries involved.

34. Reference and/or URL for published protocol.

If the protocol for this review is published provide details (authors, title and journal details, preferably in Vancouver format)

Add web link to the published protocol.

Or, upload your published protocol here in pdf format. Note that the upload will be publicly accessible.

No I do not make this file publicly available until the review is complete

Please note that the information required in the PROSPERO registration form must be completed in full even if access to a protocol is given.

36. Keywords.

Give words or phrases that best describe the review. Separate keywords with a semicolon or new line. Keywords help PROSPERO users find your review (keywords do not appear in the public record but are included in searches). Be as specific and precise as possible. Avoid acronyms and abbreviations unless these are in wide use.

37. Details of any existing review of the same topic by the same authors.

If you are registering an update of an existing review give details of the earlier versions and include a full bibliographic reference, if available.

38. * Current review status.

Update review status when the review is completed and when it is published.New registrations must be ongoing so this field is not editable for initial submission.
Please provide anticipated publication date

Review_Ongoing

40. Details of final report/publication(s) or preprints if available.

Leave empty until publication details are available OR you have a link to a preprint (NOTE: this field is not editable for initial submission). List authors, title and journal details preferably in Vancouver format.

Give the link to the published review or preprint.
