# Supplementary material for: Social Media Use and Health and Well-being of Lesbian, Gay, Bisexual, Transgender, and Queer Youth: Systematic Review
Source: J Med Internet Res. 2022 Sep 21;24(9):e38449. doi: 10.2196/38449 (PMC9536523; doi:10.2196/38449)
Supplement: Multimedia Appendix 2 [file jmir_v24i9e38449_app2.docx]

**Search Strategy**

***CINAHL***

"LGB*" or "GLB*" or (MH "Sexual and Gender Minorities+") or (MH "Gay Persons+") or (MH "Lesbians") or "queer" or (MH "Transgender Persons+") or "sexually and gender diverse" or "gender and sexuality diverse" or "homosexual*" or "bisexual*" or (MH "Sexual Orientation+")

AND

"identit*" or "support*" or "help*" or "friend*" or "relationship*" or "partner*" or (MH "Mental Health") or (MH "Depression+") or (MH "Anxiety+") or "mood disorder" or (MH "Stress Disorders, Post-Traumatic+") or "ptsd" or "suicid*" or (MH "Injuries, Self-Inflicted") or "wellbeing"

AND

(MH "Social Media+") or "social networking site*" or (MH "Facebook") or "instagram" or "tumblr" or (MH "Twitter") or "YouTube" or "LinkedIn" or "WeChat" or "Snapchat" or "TikTok"

AND

"adolescen*" or "young adult*" or "teen*" or "youth*"

***OVID Embase***

LGB*.mp. or GLB*.mp. or (Sexual and Gender Minorities).mp. or gay.mp. or lesbian.mp. or queer.mp. or transgender.mp. or (sexually and gender diverse).mp. or (gender and sexually diverse).mp. or homosexual*.mp. or bisexual*.mp. or sexual orientation.mp.

AND

identit*.mp. or support*.mp. or help*.mp. or friend*.mp. or relationship*.mp. or partner*.mp. or exp mental health/ or depression/ or exp anxiety/ or exp mood disorder/ or exp posttraumatic stress disorder/ or PTSD.mp. or suicid*.mp. or self-harm.mp. or exp wellbeing/

AND

social media*.mp. or social networking site*.mp. or Facebook.mp. or Instagram.mp. or Tumblr.mp. or Twitter*.mp. or YouTube.mp. or LinkedIn.mp. or WeChat.mp. or Snapchat.mp. or TikTok.mp.

AND

adolescen*.mp. or young adult*.mp. or teen*.mp. or youth*.mp.

***OVID MEDLINE***

LGB*.mp. or GLB*.mp. or (Sexual and Gender Minorities).mp. or gay.mp. or lesbian.mp. or queer.mp. or transgender.mp. or (sexually and gender diverse).mp. or (gender and sexually diverse).mp. or homosexual*.mp. or bisexual*.mp. or sexual orientation.mp.

AND

identit*.mp. or support*.mp. or help*.mp. or friend*.mp. or relationship*.mp. or partner*.mp. or exp mental health/ or exp depression/ or exp anxiety/ or exp mood disorder/ or exp posttraumatic stress disorder/ or PTSD.mp. or suicid*.mp. or self-harm.mp. or exp wellbeing/

AND

social media*.mp. or social networking site*.mp. or Facebook.mp. or Instagram.mp. or Tumblr.mp. or Twitter*.mp. or YouTube.mp. or LinkedIn.mp. or WeChat.mp. or Snapchat.mp. or TikTok.mp.

AND

adolescen*.mp. or young adult*.mp. or teen*.mp. or youth*.mp.

***Web of Science***

TS=(LGB* OR GLB* OR Sexual and Gender Minorities OR gay OR lesbian OR queer OR transgender OR sexually and gender diverse OR gender and sexually diverse OR homosexual* OR bisexual* OR sexual orientation) AND TS=(identit* OR support* OR help* OR friend* OR relationship* OR partner* OR mental health OR depression OR anxiety OR mood disorder OR posttraumatic stress disorder OR PTSD OR suicid* OR self-harm OR wellbeing) AND TS=( social media* OR social networking site* OR Facebook OR Instagram OR Tumblr OR Twitter* OR YouTube OR LinkedIn OR WeChat OR Snapchat OR TikTok) AND TS=(adolescen* OR young adult* OR teen* OR youth*)

***ACM Digital Library***

LGB* or GLB* or Sexual and Gender Minorities or gay or lesbian or queer or transgender or sexually and gender diverse or gender and sexually diverse or homosexual* or bisexual* or sexual orientation AND identit* or support* or help* or friend* or relationship* or partner* or mental health or depression or anxiety or mood disorder or posttraumatic stress disorder or PTSD or suicid* or self-harm or wellbeing AND social media* or social networking site* or Facebook or Instagram or Tumblr or Twitter* or YouTube or LinkedIn or WeChat or Snapchat or TikTok AND adolescen* or young adult* or teen* or youth*
