# Supplementary material for: Social Media Use and Health and Well-being of Lesbian, Gay, Bisexual, Transgender, and Queer Youth: Systematic Review
Source: J Med Internet Res. 2022 Sep 21;24(9):e38449. doi: 10.2196/38449 (PMC9536523; doi:10.2196/38449)
Supplement: Multimedia Appendix 4 [file jmir_v24i9e38449_app4.docx]

**Multimedia Appendix 4.** Summary of the included qualitative studies (N=18).

| Study, year, and country | Purpose | Age (years) | Sample size, N | LGBTQ^a^ sample | Method | Findings and themes | Summary and example extracts/quotes |
| --- | --- | --- | --- | --- | --- | --- | --- |
| Bates et al [27], 2020, United Kingdom | Explore LGBTQ+ youth constructing identities via social media. | 19 to 23 (mean 20.29, SD1.4) | 17 | Bisexual (n=4), nonbinary (n=1), pansexual/fluid/queer (n=3), homosexual (n=3), heterosexual (n=6) | Recruitment: email advertisements to students in small public university.  Data collection: semi-structured interviews.  Data analysis: thematic analysis. | Narratives of Merging Safe Spaces Offline/Online: feeling safe was vital for youth to express their identity. Web-based spaces are usually safe for LGBTQ youth in part due to privacy settings.  Narratives of External Identity Alignment: Some felt that LGBTQ+ labels did not fit their identity and wanted to be known for “who I already am”.  Narratives of Multiple Context-Based Identities: LGBTQ youth may identify with multiple overt identities across web-based and offline audiences.  Narratives of Individuality and Autonomy: Social media was recognized as a space unconstrained and allowed LGBTQ youth to develop identity. | Participant experiences demonstrated how social media is a transformative tool with platforms being used for differing LGBTQ identity development.  Quote: *“lots of people putting their own experiences forward, and that makes room for validation and self-discovery.”*  Quote: *“I’ve just started saying that I’m queer which covers everything, … not to say I am, it just covers all grounds.”* |
| Byron et al [31], 2019, Australia | How Tumblr is used among LGBTQ youth to connect with peers, develop identity and wellbeing. | 16 to 34 (mean 24.6) | 23 | transgender/nonbinary (n=11), homosexual (n=8), bisexual (n=5), queer (n=6), asexual (n=3), pansexual (n=3). | Recruitment: via social media advertisements and flyers to LGBTQ organizations.  Data collection: semi-structured interviews; questionnaires/surveys.  Data analysis: not described. | Queer Tumblr: Tumblr offers a safe space for gender diverse and transgender youth which does not rely on existing relationships.  Lessons on Gender and Sexuality: Tumblr is an effective tool for identity development where experiences are shared, and affirmation is achieved.  Communities, Followers, and “People Like Me”: Sharing experiences was not indicative of needing a response but rather to be heard.  “Somewhere to Put Things”: Recording, Documenting, and Processing Queer Lives: Tumblr acted as a curatorial space for documenting life experiences (e.g., gender transitioning).  “Everything Was Problematic”: Tumblr Intensities: Tumblr commonly transformed from an identity tool to a negative space.  Leaving Tumblr: Negativity caused many participants to limit use or leave. | Tumblr as a social media platform allowed LGBTQ users to exchange experiences, explore sexuality and gender identity, and peer support.  Quote: *“I actually learned about agender and … other genders from Tumblr. Before that, all I really knew was there are men and there are women, … I engage with … nonbinary people and trans people quite a bit.”*  Quote: *“[Tumblr] introduced me to the concept of asexuality. Stopped [using] because Tumblr is 90% toxic cesspool now, sadly”* |
| Craig and McInroy [32], 2014, Canada (Toronto) | Examine self-disclosure and identity development of LGBTQ youth in online media. | 18 to 22 (mean 19.47, SD 1.2) | 19 | Gay (n=6), lesbian (n=4), bisexual (n=2), polysexual (n=1), queer (n=1), transgender/genderqueer (n=4), transgender/transsexual male (n=3), genderqueer (n=1), cisgender (n=15) | Recruitment: email advertisements to LGBTQ organizations.  Data collection: semi-structured interviews.  Data analysis: grounded theory. | Coming Out Digitally: Social media was commonly used to disclosure LGBTQ identity however, required considerable effort in assessing audiences’ reactions prior. | LGBTQ youth were able to access new media including social media to explore their identity and disclose their identity. Participants were also able to use this function to extend their identities offline.  Quote: *“I think the big thing about coming out now is … Facebook interested or Facebook in a relationship. That’s a big decision because everybody on your Facebook list is going to see that.”* |
| Duguay [47], 2016, United Kingdom | Explore LGBTQ youths’ decisions on self-disclosure and context collapse and its prevention. | 18 to 25 (mean 20) | 27 | Gay (n=14), bisexual (n=5), lesbian (n=4), queer (n=2), pansexual (n=1), asexual (n=1) | Recruitment: from LGBTQ groups at 11 universities.  Data collection: semi-structured interviews.  Data analysis: grounded theory. | Identity Disclosures and Experiences of Context Collapse: Few participants disclosed identity with high visibility as a timeline post, more commonly indirect methods such as use of Facebook’s ‘interested in’ function. Some displays of LGBTQ identity leaked into unintended audiences via ‘likes’, group memberships, friends’ posts or photos.  Strategies for Preventing Context Collapse: To prevent unintended identity disclosure audiences were separated taking significant effort to ‘weed out’ contacts. | LGBTQ identity disclosure was influenced by the design of social media platforms. Individuals would reinstate heteronormative appearance with particular audiences to avoid accidental disclosure.  Quote: *“I don’t want to run the risk of having a confrontation in real life so if I have it on Facebook, they can take it in … and then it’s over and done with and I never had to say anything.”* |
| Fox and Ralston [48], 2016, United States (Midwest) | Identify learning experiences of LGBTQ youth on social media, and how social media shapes the experiences. | 18 to 28 (mean 20.91, SD 2.65) | 33 | Gay (n=8), lesbian (n=4), bisexual (n=13), transgender (n=4), transgender (n=4), asexual (n=2), genderqueer (n=2), pansexual (n=2) | Recruitment: flyers displayed on community boards targeting LGBTQ participants in a large city.  Data collection: semi-structured interviews.  Data analysis: grounded theory/thematic coding. | Traditional Learning: Social media was a tool used to learn about identity terminology and meaning, transitioning and community connections.  Experiential Learning: Participants described methods to explore identity before disclosing identity (eg, sharing/posting support for LGBTQ rights on social media).  Teaching Others: Once identity was established participants shared LGBTQ information or engaging in discussions within their networks. | Social media allowed LGBTQ youth to visualize via posts similar identifying peers thus assisting their identity development. Participants found it important to seek peers in similar circumstances and experiences.  Quote: *“Before I joined Tumblr, I felt like I didn’t have language for a lot of things, like I didn’t know transgender was a thing until then. So, knowing about those things definitely helped the process.”* |
| Hanckel et al [49], 2019, Australia | How identity is developed and managed across social media. | 16 to 34 (mean 24.6) | 23 | Lesbian (n=3), gay (n=5), bisexual (n=5), asexual (n=3), pansexual (n=2), panromantic (n=1), queer (n=6), transgender (n=7), nonbinary (n=3), agender (n=1), genderfluid (n=2) | Recruitment: not described.  Data collection: in-depth interviews.  Data analysis: not described. | Curation of LGBTIQ+^b^ Identity Across Social Media Spaces: Participants were able to manage the degree of anonymity across platforms and select audiences to share LGBTQ content with. There is emotional labor involved in ‘unfriending’ or blocking people and removing existing LGBTQ content.  Platforms, Policies and Normative Space/s: Curation Constraints and Possibilities: Gender-diverse participants were able to change names with select contacts (ie, via Facebook Messenger). Many shared or sought LGBTQ information. | LGBTQ young adults were able to utilize social media to seek and foster support. LGBTQ participants negotiated risk on social media utilizing platform specific functions (e.g., unfollowing, blocking and anonymity).  Quote: *“It helps … that I’m anonymous, I feel a lot more open … about my sexuality on Instagram.”* |
| Harper et al [33], 2016, United States (Chicago and Miami) | Explore the internet’s role in development of sexually diverse identities. | 15 to 23 (mean 19) | 63 | Gay (n=46), bisexual (n=15), questioning (n=2) | Recruitment: same-sex attracted males from larger pool.  Data collection: semi-structured interviews.  Data analysis: phenomenological inquiry framework. | Learning About and Exploring Sexual Orientation and the Gay Community: Exploring sexuality web-based affirmed identity and learn through peer narratives.  Connecting and Socializing with Other Gay and Bisexual Peers: Internet including social media was a widely used tool for connecting with LGB peers.  Gaining Self-Acceptance and Sharing Sexual Orientation Identity with Others: web-based and offline LGB^c^ networks provided youth with emotional comfort. The internet was also used for self-disclosure. | Participants found that the internet including social media offered a space for identity exploration and acceptance. Participants achieved this from increased awareness, learning about gay/bisexual communities, connecting with other gay/bisexual men, and self-disclosure.  Quote: *“Through the internet, these affirmations served a positive role in helping youth to come to terms with their sexual orientation identity.”* |
| Herrera [50] 2018, location not reported | Explore the relationship between hashtags and lesbian identity. | 18 to 30 (mean 24.15) | 20 | Lesbian (n=15), gay (n=3), bisexual (n=1), queer (n=3), fluid (n=1) | Recruitment: commenting on Instagram posts of those using lesbian-related hashtags.  Data collection: semi-structured interviews  Data analysis: grounded theory. | What is a lesbian? #lesbian could be used to display sexual desire or sexuality among followers.  “Labels Suck”: The Social Imperative to Claim an Intelligible Sexual Identity: Most shared dislike for sexual identity labels finding them too restrictive.  Hashtagging #lesbian: The Technological Imperative to Name the Sexual Self: #lesbian can be searched allowing users to view public profiles however, for this function labels must be used.  (#)Queer: A Viable Alternative to (#)lesbian? #queer could be used in addition to #lesbian expressing wide sexual and gender identities. Label use was used strategically or when needed. | Instagram as a social media platform allows a user to express identity through words as hashtags (which enable audiences to view their images). The hashtag allows the user to construct an identity and encourage others to account for their own identity.  Quote: *“lesbian can be a bit limiting and simplified.”* |
| Hillier et al [28], 2012, United States | Explore LGB youth internet use for social support, friendships and romantic relationships compared to non-LGB youth. | 13 to 18 | 59 | LGB (n=33), non-LGB (n=26) | Recruitment: email advertisement to gay, lesbian, and straight organizations.  Data collection: focus groups.  Data analysis: thematic analysis. | Online Friendships: ‘Perv’, ‘stalker’ and ‘serial killer’ were common words used by non-LGB youth to describe connecting with online strangers. Whereas online connections were more accepted by LGB youth.  Support from Friends Online: The level of support among LGB youth was likely subject to their disclosure status offline. LGB youth received support online and used it to come out usually before doing so offline.  Finding Romance Online: Some LGB youth formed online relationships from social media. | LGB adolescents utilized the internet including social media for connecting with LGB communities and social support. Most participants sought to explore sexual attraction and feelings.  Quote: *[Non-LGB] “I don’t meet new people online in case they are kidnappers.”*  Quote: *“My ex-boyfriend, I met him on*  *Myspace and went out with him.”* |
| Lucero [29], 2017, Ukraine and United States | Examine whether social media provides LGBTQ youth a safe space for identity exploration and expression. | 14 to 17 (mean 16.3) | 19 | Lesbian (n=3), gay (n=8), bisexual (n=1), queer (n=1), unsure (n=3), not straight (n=3) | Recruitment: flyers sent to LGBTQ organizations and Facebook.  Data collection: questionnaires/surveys.  Data analysis: social constructionist theory. | Comfort Online: Participants felt that support was more accessible on social media and felt safe compared to offline. | LGBTQ youth felt comfortable expressing their LGBTQ identity on social media as a safer space than offline alternatives.  Quote: *“On Facebook, I am more likely to find support.”* |
| McConnell et al [51], 2018, United States (Chicago) | Examine the relationship between Facebook and LGBTQ youth identity management. | 19 to 28 (mean 24.13, SD 1.64) | 49 | Identifying as male (n=77), identifying as female (n=108), transwomen (n=15), transmen (n=3), gay (n=69), lesbian (n=55), bisexual (n=49), heterosexual (n=10), unsure (n=8) | Recruitment: LGBTQ youth from a longitudinal study.  Data collection: open-ended interviews.  Data analysis: not described. | Qualitative Experiences Online: Participants had varying levels of disclosure among on- and offline networks. Facebook could be used to manage how LGBTQ youth express identity by using multiple accounts for different audiences or censoring what they share. Additionally, Facebook allowed for affirmation via ‘likes’ on posts. | LGBTQ youth free self-expression on social media is complicated due to factors relating to identity disclosure. By investigating Facebook accounts, youth were mostly either categorized as low outness or high outness. Some would purposely censor their identity expression to avoid unintentional identity disclosures.  Quote: *“[My female partner] and I recently got married, so we’ve been posting some pictures … People are very supportive of our relationship and enjoy looking at our pictures.”* |
| McInroy et al [53], 2015, Canada (Toronto) | Investigate media representation of transgender individuals (including social media). | 18 to 22 (mean 19.47, SD 1.2) | 19 | Gay (31.6%), lesbian (21.1%), bisexual (10.5%), queer/polysexual (10.6%), cisgender (79%), transgender man (15.8%) | Recruitment: from organizations as part of a larger study.  Data collection: semi-structured interviews.  Data analysis: grounded theory. | Online Media: Explicit Transphobia Versus Resources that Support Healthy Development: Social media is a resource that promotes health development of identity among transgender youth. It serves as a wealth of information about transgender identity (e.g., transitioning). | Transgender youth were able to utilize social media to construct support networks among transgender peers. These networks reported experiences of transitioning and encouraged a feeling of connectedness.  Quote: *“I’d just go … [to] YouTube and type in transgender and then some guys that were trans[gender], they’d pop up. And then they’d have videos of their whole [transition] process”* |
| Paceley et al [54], 2020, United States (Midwest) | Explore how LGBTQ youth in rural areas use social media for identity development. | 14 to 18 (mean 16) | 34 | Gay (21%), lesbian (12%), bisexual (32%), queer (3%), transgender (12%), cisgender (79%) | Recruitment: advertisements on social media and flyers, part of a larger study.  Data collection: semi-structured interviews.  Data analysis: grounded theory. | Finding Community: Social media is a space where LGBTQ youth find peers, especially those living in rural areas.  Expressing Oneself: Social media was a method LGBTQ youth used for identity disclosure and venting using anonymity strategically.  Seeking Resources and Information: Social media connections allowed for identity support, particularly Tumblr. | LGBTQ adolescents used social media to construct networks among other LGBTQ individuals. Some specifically referenced the lack of LGBTQ networks in rural and remote locations. These networks were further used to create friendships and share identity specific experiences.  Quote: *“Just full of gay people. Tumblr is the gay person’s haven.”* |
| Rubin et al [34], 2015, United States (San Francisco) | Examine identity management on Facebook among LGBTQ youths and effect on emotional health and social support. | 16 to 19 | 8 | Lesbian (n=5), bisexual (n=3) | Recruitment: web-based advertisements via websites and social media.  Data collection: semi-structured interviews.  Data analysis: thematic analysis. | Emotional Labor of Concealment: Offline monitoring of heteronormativity extended to Facebook.  Facebook and Homophobia: Facebook was a space for LGBTQ youth to view LGBTQ content or express identity with the limitation of witnessing homophobia.  Labor of Social Surveillance: Significant labor was reported in mitigating stigma through censoring Facebook content.  Ruminating About Profile Content: Continuing monitoring of Facebook content was emotionally laborious. | Presence of homophobia and heteronormative attitudes has made sexually diverse individuals feel a lack of belongingness. Identities were managed online to prevent unintentional disclosure and remain 'virtually closeted'. Participants described symptoms of depression and anxiety when monitoring their social media due to fear of being outed and excluded.  Quote: *“I don’t mark my sexual orientation on Facebook partly because … there is hatred against gays and lesbians in the USA, in my school, and with my parents.”* |
| Selkie et al [56], 2020, United States (Midwest) | Understand transgender youths’ uses of social media for support. | 15 to 18 (mean 16) | 25 | Transfeminine (n=11), transmasculine (n=13), nonbinary (n=1) | Recruitment: from gender services clinic.  Data collection: semi-structured interviews.  Data analysis: thematic analysis. | Emotional Support: Social media is a space to interact with other transgender youth.  Appraisal Support: Transgender representation on social media provided transgender youth validation.  Informational Support: Transgender youth were able to access information such as transitioning therapies and surgery. This was also beneficial in informing parents of transgender youth.  Negative Social Media Experiences: Social media is a positive experience for most but is prone to witnessing/experiencing negative interactions. | Communities of support can be formed via social media among transgender adolescents which provide appraisal, information, and emotional support. Web-based networks can be subject to exclusionary behaviors and harassment.  Quote: *“Social media … there's more anonymity there really, more people are open to talking about their experiences as trans people and helps you understand it more.”* |
| Singh [57], 2013, United States (Southeast) | Explore resilience strategies among transgender youth of colour negotiating prejudice and racism. | 15 to 24 (mean 18.3) | 13 | Transgender male (n=5), transgender female (n=4), transsexual female (n=1), genderqueer (n=2), genderfluid (n=1) | Recruitment: flyers distributed to organizations servicing transgender youth.  Data collection: semi-structured interviews.  Data analysis: grounded theory. | Use of Social Media to Affirm One’s Identities as a Transgender Youth of Color: Participants connected via various platforms (ie, Facebook, Myspace, Twitter, and trans-specific platforms) which provided inspirational transgender role models. | Social media has been associated with connecting with transgender peers of color to affirm identity. Transgender youth also used these networks to understand connections between ethnicity and their gender identity.  Quote: *“[Participant] wished there was more access at school to trans sites — it’s cool to have people to look up to and follow on Twitter.”*  Extract: *“Social media helped [participants] see new perspectives — namely transgender-positive and racial/ethnic-affirming ones.”* |
| Taylor et al [58], 2014, United Kingdom (Newcastle, Manchester, and London) | Understand LGBTQ youth negotiate Christian and sexually and gender diverse identities. | 17 to 34 (mean 24) | 38 | Gay (n=15), lesbian (n=13), bisexual (n=5), queer (n=4), asexual (n=1), genderqueer (n=3), transgender (n=1), transsexual (n=1) | Recruitment: web-based via the study’s website and Facebook.  Data collection: semi-structured interviews.  Data analysis: thematic analysis. | ‘Coming Out’ as Queer and Religious Online – Negotiating (Dis)Embodied Identities: Social media (i.e., Facebook and Twitter) provided religious youth opportunities to disclose LGBTQ identity online. Some will deliberately hide their LGBTQ identity from what religious groups can view.  (Dis)Embodiment, (Dis)Connection and Temporality: Certain factors illuminate a particular identity (religious or LGBTQ) depending on activities.  Online Spaces, New Opportunities? Social media allows youth to negotiate religious and LGBTQ identities. | Participants described social media such as Facebook beneficial to allowing a smoother disclosure of sexual/gender identity and being religious. Social media can offer a space to negotiate identities for LGBT religious youth.  Quote: *“On Facebook …, you’ve got a little box to fill in a brief description of you, their religious views and sexual orientation going to go in there definitely.”* |
| Varjas et al [60], 2013, United States (Southeast) | Explore LGB youths’ perceptions of technology use in relation to cyberbullying and cybervictimization. | 15 to 18 (mean 17.1, SD 0.9) | 18 | Lesbian (n=5), bisexual (n=3), gay (n=9) | Recruitment: from organizations serving LGB youth.  Data collection: semi-structured interviews.  Data analysis: grounded theory. | Constructive Technology Use by LGB Adolescents: Social media provides youth a comfortable method to explore sexual identities using anonymity. Youth could connect other LGBTQ individuals and transition to offline friendships or romantic relationships. | Technology including social media was beneficial to LGB youth particularly those feeling depressed and isolated. Participants also used these tools to seek social support and identity disclosure.  Quote: *“MySpace and Facebook … might be the only way and it’s like a safeguard [be]cause they might not know you on the Internet and so it’s like a safe haven, a place to be yourself.”* |

^a^LGBTQ: lesbian, gay, bisexual, transgender, and queer.

^b^Lesbian, gay, bisexual, transgender, queer, intersex, and other non-heterosexual and gender diverse

^c^LGB: lesbian, gay, and bisexual.
